# Acupuncture combined tuina for oculomotor paralysis: A protocol for systematic review and meta-analysis

**DOI:** 10.1097/MD.0000000000028456

**Published:** 2022-01-14

**Authors:** Yan Huang, Zuoxiong Miao, Chunbai Lai, Fanhua Zeng, Qin Guo, Tonghai Zhang, Mingheng Li, Zhenhua Xu

**Affiliations:** a The Second Clinical School, Guangzhou University of Chinese Medicine; b Department of Acupuncture Rehabilitation, Ganzhou Hospital of Chinese Medicine; c Department of Spine Surgery, Ganzhou People's Hospital; d Guangdong Provincial Hospital of Chinese Medicine, Guangzhou, Jiangxi, China.

**Keywords:** acupuncture, oculomotor paralysis, protocol, systematic review, tuina

## Abstract

**Background:**

Oculomotor paralysis (OP) is a neurologic syndrome with multiple causes of oculomotor nerve and its dominant tissue and muscle dysfunction. Acupuncture combined with tuina is a wide-ranging used rehabilitation therapy, although there is short of supporting evidence for its efficacy and safety in patients with OP. The purpose of this systematic review was to estimate and synthesize evidence of the efficacy and safety of acupuncture combined with tuina in the treatment of OP.

**Methods:**

Electronic databases, including PubMed, Web of Science, Cochrane Library, EMBASE, Technology Journal and China Science, China National Knowledge Infrastructure, Chinese Biomedical Literature Database, and Wanfang,

adopt an appropriate search strategy. RevMan V.5.3.5 software will be used for data synthesis, bias risk, and subgroup analyses.

**Results:**

This study provides high-quality evidence to assess the effectiveness and safety of acupuncture combined with tuina for OP.

**Conclusion:**

This systematic review explores whether acupuncture combined with tuina is an effective and safe intervention for OP.

**Ethics and dissemination:**

Private information from individuals will not publish. This systematic review does not involve endangering participant rights. Ethical approval was not obtained. The results may be published in a peer-reviewed journal or disseminated at relevant conferences.

**PROSPERO registration number:**

PROSPERO CRD42021266447

## 1 Introduction

Oculomotor paralysis (OP) is a neurological syndrome with multiple causes of oculomotor nerve and its dominant tissue and muscle dysfunction, which has a far-reaching negative impact on everyday life, and is an uncommon event in the general population. In one-third of OP cases, the cause is unknown, and the common causes for this condition include cerebral aneurysm, tumors, skull trauma and its sequelae, virus, inflammatory infections, diabetic neuropathy, or vascular processes.^[1–3]^ Their estimated percentages were approximately the following values: Congenital (20–25%); Trauma (20%); Vascular ischemia (15%); Tumors (10–20%). The remainder responds to a variety of causes, such as aneurysm, metabolism, infection or by-infection, and degenerative.^[4]^

A wide range of treatments have been used to cure OP, such as methycobal, vitaminB12, and tetracycline. Treatment is targeted to relieve the source of the palsy; in cases in which the cause is unknown, the treatment is mostly supportive.^[5–8]^ All conservative treatments after OP were discontinued. However, all of them have adverse effects and unsatisfactory efficacy, and with the increasing popularity of complementary and alternative medicine in the general population, patients are looking for options other than conventional treatment.^[9]^

Acupuncture therapy, which is widely used for a long time in some Asian countries, is a major part of traditional Chinese medicine.^[10]^ Previous studies have shown that acupuncture can be applied in many nervous system diseases, such as stroke, vascular mentia, and facial paralysis.^[11–13]^ Many clinical studies have proven that acupuncture is effective and has no adverse effects, and systematic evaluation shows that acupuncture is effective and safe in the treatment of OP.^[14]^ The most commonly used acupuncture points for OP are Cuanzhu (BL2), Jingming, Chengqi (ST1)(BL1), Sibai (ST2), and Taiyang (EX-HN5), and the most commonly used auricular points are yan (LO5), shen (CO10), and gan (CO12).^[15]^ The acupoints around the eyes were mainly punctured to promote the flow of qi and blood, nourish the muscles and tendons, and improve the circulation of qi and blood around the eyes,^[16]^ which can promote eyeball movement and recover the function of ocular muscles.^[2,17]^

Tuina is an ancient external treatment method.^[18]^ It is based on the TCM theory of meridians and acupoints, and uses specific operation skills to act on the patient's body surface or acupoints to treat the disease,^[19,20]^ which has the characteristics of intervention and low final cost, for OP; at present, there is no system assessment of tuina in the treatment of OP, so this study will assess the effectiveness and safety of tuina in the treatment of OP, and provide a basis for clinical massage decisions; acupuncture and tuina are part of the external treatment of TCM, and both are based on the same theory of meridians and acupoints. The use of traditional Chinese medicine acupuncture combined with tuina therapy for patients with OP can effectively improve clinical symptoms and enhance patients’ quality of life, and is recommended for clinical use and active promotion.

## 2 Methods

### 2.1 Study registration

This systematic review protocol was registered on PROSPERO as CRD42021266447. The protocol followed the Cochrane Handbook for Systematic Reviews of Interventions and the Preferred Reporting Items for Systematic Reviews and Meta-Analysis Protocol (PRISMA-P) statement guidelines.^[21]^ We describe the changes in our full review, if needed.

### 2.2 Inclusion criteria for study selection

#### 2.2.1 Types of studies

The review will include randomized controlled trials (RCTs) that were reported in English or Chinese without any regional restrictions. First period of randomized cross-over trials were included. Non-RCT reviews, animal experimental studies, case reports, expert experience, conference articles, and duplicated publications will be removed.

#### 2.2.2 Types of participants

People with a diagnosis of OP will participate without considering any information related to their age, race, nationality, education, sex, or economic status; patients with OP are between the ages of 18 and 70 years. There will be no race or other restrictions and participants of any sex and ethnicity will be enrolled.

#### 2.2.3 Types of interventions

All kinds of acupuncture and tuina will be included. In the experimental groups, we plan to include the following acupuncture therapies: body acupuncture, manual acupuncture, dermal needle, electroacupuncture, auricular acupuncture, ocular acupuncture, scalp acupuncture, fifire needling, plum blossom needle, warm needling, tuina, and massage. Pharmacoacupuncture, laser acupuncture, acupoint injection, cupping, moxibustion, and transcutaneous electrical nerve stimulation will be excluded. In addition, there were no limitations to the test cycle and treatment frequency.

#### 2.2.4 Types of outcome measures

##### 2.2.4.1 Primary outcome

The primary outcome measurement will be an improvement in the scores of diplopia, pupillary light reflex, and size of palpebral fissures. The pupillary light reflex was measured using the visual analog scale (VAS). The pupillary light reflex will be assessed by observing the changes in pupil size in light and dark places before and after treatment, and the size of the palpebral fissure was measured from the midpoint of the upper eyelid to the midpoint of the lower eyelid.

##### 2.2.4.2 Secondary outcome

The secondary outcomes of the review will include as follows:

1. Measure the patient's quality of life: Measure the patients’ quality of life: Quality of life assessment scale SF-36(short form 36 questionnaires).
2. Compare pupil diopter: Compare pupil diopter: record the distance when the patient can see the thing before and after treatment under the same light intensity.
3. The adverse effects.

### 2.3 Exclusion criteria

1. Republished papers.
2. Articles published as abstracts or with incomplete data, or complete articles could not be obtained even after contacting the author(s).
3. Studies exhibiting a high-risk bias.
4. Experiences, letters, animal experiments and systematic reviews.
5. Acupuncture combined with tuina was not only in the experimental group but also in the control group.
6. Articles without full text or with data which are missed or cannot be used.

### 2.4 Search methods for identification of studies

#### 2.4.1 Electronic searches

The following databases will be searched from their inception until July 2021: MEDLINE, Embase, Allied and Complementary Medicine Database, Cochrane Central Register of Controlled Trials, Chongqing VIP Chinese Science, China National Knowledge Infrastructure and Technology Periodical Database, SinoMed, China Doctoral Dissertations Full-text Database, Wanfang Database, and the China Master's Theses Full-text Database. Only RCTs that investigate the effects of acupuncture combined with tuina for OP in comparison with the aforementioned comparator controls will be included. Chinese translations of these search terms will be used in Chinese databases.

#### 2.4.2 Searching strategy

The search strategy for the Web of Science (WOS) database is shown in Table 1, which includes all the search terms. The same strategy is used in other electronic databases.

**Table 1:** Web of science search strategy.

Number	Search terms
1	Randomized controlled trial
2	Randomized clinical trial
3	Controlled clinical trial
4	Randomly
5	Randomized
6	Trial
7	Or/1–6
8	Oculomotor paralysis
9	Oculomotor nerve palsy
10	BlepharoptosParalysis of oculomotor nerveis
11	Blepharoptosis
12	Ptosis
13	Eyelid Ptosis
14	OMNP
15	Third nerve palsy
	Or/8–15
	acupuncture
	Chinese tuina
	electroacupuncture
	electro-acupuncture
	auricular acupuncture
	acupoint
	Tuina
	Chinese tuina
	Massage
	Chinese massage
16	Or/17–26
17	7,16 and 27

### 2.5 Data collection and analysis

#### 2.5.1 Selection of literature

**Figure 1:**
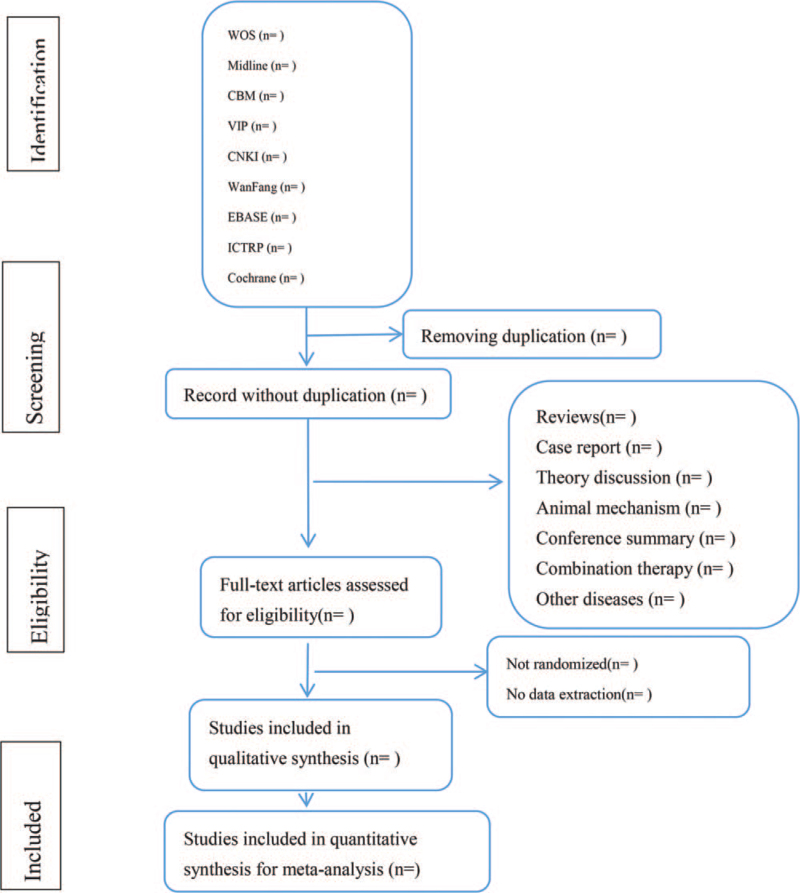
Flow diagram of studies identified.

Two researchers will independently determine the study according to the inclusion criteria: First, duplicate studies were removed using EndNote software (V.x9.0). Second, screening the headline and abstract, if necessary, read the full article to confirm if it should be included. They also used EndNote software to manage the included studies. The screening operation is performed as shown in Figure 1. If there is disagreement during the screening process, we discuss with the third expert to make a decision.

#### 2.5.2 Data extraction and management

Two authors independently extracted the data from the included studies. In multiarm RCTs, we will extract data from RCTs of 2 arms, while we will select 1 group, which contains the treatment of acupuncture combined tuina as the treatment group, we will also choose another group the treatment of which without acupuncture combined tuina as the control group. The general information consisted of title, authors, country, publication year, language, and journal source; information of participants: gender, age, sample size, and duration of onset; information on intervention characteristics: type, session, duration, and follow-up time; and outcome information about primary outcome, second outcome, view time points, and adverse reactions.

#### 2.5.3 Assessment of risk of bias in included studies

Two independent authors evaluated the risk of bias using the Cochrane Collaboration bias risk assessment tool to assess the risk bias of the literature included in the systematic review. The 2 authors assessed the risk of bias in sequence generation, allocation concealment, incomplete outcome data, blinding of participants personnel, outcome assessment, selective outcome reporting, and other biases. The evaluation grades were low, high, and unclear risk of bias.

#### 2.5.4 Dealing with missing data

When the included article lacks some important information, we will try to contact the communication author by e-mail, phone, or other contacts. If we cannot obtain the information through the methods above, we will turn to the following strategies to evaluate the potential influence of missing data.

#### 2.5.5 Assessment of heterogeneity

We used the Q-test and *I*^2^ statistic to assess the heterogeneity of the included studies, as the criteria: *I*^2^*<*50% indicated low heterogeneity, while *I*^2^ >50% indicated high heterogeneity.

#### 2.5.6 Data synthesis

The meta-analysis of intervention and outcome measures methods will be conducted using RevMan V.5.3.5 software (Oxford, the Cochrane Collaboration, UK). If the statistical heterogeneity is high (*P* < .1, or *I*^2^ > 50%), we will use the random-effect model, while if the statistical heterogeneity is low (*P* > .1, or *I*^2^ < 50%), we will use the fixed-effect model to combine the data. However, if the level of heterogeneity is significant, descriptive statistics will be performed.

#### 2.5.7 Subgroup analysis and investigation of heterogeneity

If we find substantial heterogeneity in the included studies, subgroup analysis will be conducted according to race, sex, age, types of acupuncture combined with tuina, sample size, and other factors.

#### 2.5.8 Sensitivity analysis

We conduct a sensitivity analysis to assess the robustness and reliability of the combined results. If the results are not stable, we may turn to removing studies with high risk of bias, or cheek up processing method of missing data (worst-case scenario analysis: all participants with missing data were counted as failures; extreme worst case/best-case scenario analysis: participants with missing outcome data).

#### 2.5.9 Grading of evidence quality

We will use the Development and Evaluation to Grading of Recommendations Assessment to access confidence in cumulative evidence.^[22]^ Risk of bias, indirectness, imprecision, heterogeneity, and publication bias will be assessed, and the results will be divided into 4 levels: high, moderate, low, and very low.

#### 2.5.10 Assessment of reporting bias

We constructed funnel plots to assess asymmetry only if at least 10 RCTs were included.

#### 2.5.11 Ethics and dissemination

Ethical approval was not required in this study because there is nothing of the data that has a relationship with an individual patient. This systematic review will be completed according to PRISMA guidelines. This review will provide an assessment of the effect and safety of acupuncture combined with tuina for OP, and we will publish the findings in a peer-reviewed, open-access journal, and the completed systematic review and meta-analysis will be disseminated online, which would be obtained freely for anyone. These results may contribute to improving the therapeutic strategy for patients with OP.

## 3 Discussion

OP is a disease caused by a variety of causes and its dominant tissue and muscle function, often manifested as ptosis, diplopia, eye activity dysfunction, and pupil changes.^[23]^ The most common causes include head trauma, tumor, and cerebrovascular diseases caused by diabetes, hypertension, and immune diseases; however, the treatment of OP includes surgical intervention, oral nutritional nerve drugs, or glucocorticoids, but has not achieved satisfactory efficacy.^[24,25]^

This systematic review will focus on the efficacy and safety of acupuncture combined with tuina for OP. Acupuncture therapy is used to treat a variety of diseases, such as stroke rehabilitation, pain, pressure sores, urinary dysfunction, etc. Many clinical researches have proved that acupuncture is effective and has no adverse effects.^[5]^ Clinical reports show that acupuncture combined with tuina is effective for OP; however, high-quality studies still do not appear. We conducted this review to provide better evidence and guidance for clinical decision-making. We plan to publish this review within 1 year after the publication of the protocol, and we will update it every three years.

## Author contributions

**Conceptualization:** Zhenhua Xu.

**Data curation:** Chunbai Lai, Fanhua Zeng, Qin Guo,Tonghai Zhang.

**Formal analysis:** Yan Huang.

**Funding acquisition:** Zhenhua Xu.

**Project administration:** Mingheng Li.

**Resources:** Mingheng Li, Zhenhua Xu.

**Writing – original draft:** Yan Huang, Zuoxiong Miao, Chunbai Lai, Fanhua Zeng, Qin Guo.

**Writing – review & editing:** Yan Huang, Zuoxiong Miao.

## References

[1] Zhou LY Ji XJ Zhao M. Progress of treatment on oculomotor paralysis with electroacupuncture. Zhongguo Zhen Jiu 2011;31:286–8. 21644327

[2] Gu ZY Luo F. Study on acupuncture for treatment of oculomotor paralysis according to syndrome differentiation of meridians. Zhongguo Zhen Jiu 2010;30:129–32. 20214071

[3] Huber A. Treatment of oculomotor paralysis. Ophthalmologica 1968;156:498–508. 5716883 10.1159/000305562

[4] Rodríguez-Sánchez JM Ruiz-Guerrero MF. Abordaje diagnóstico y terapéutico de las parálisis oculomotoras. Rev Neurol 2001;32:148–56. 11299479

[5] Tiffin PA MacEwen CJ Craig EA Clayton Q. Acquired palsy ot the oculomolnr, trochlear and ahducens nerves. I-yc 1996;10:377–84. 10.1038/eye.1996.77 8796166

[6] Malone T|, Nerad JA. The surgical treatment of blephariiptosis in oculomotor nerve pahy. Am J Ophlhalmol.:57-64.

[7] Kose S VIrtemen O Pamukcu K. An approach to the surgical management of total ocukimoior nerve palsy. Strabismus 2001;9:01–8. 10.1076/stra.9.1.1.712 11262694

[8] Frenkel M Frenkel J. Oculomotor nerve palsy treated with acupuncture. Altern Ther Health Med 2002;8:120, 118. 11890379

[9] Eiseciberg DM Davis RB Eiiner SI. Treiuls in alternative medicine use in the United Slates, 1990–1997. MM^ 1998;280:1569–75. 10.1001/jama.280.18.1569 9820257

[10] Chen Z Wang R Zhang M. Acupuncture combined with medication for opioid use disorder in adults: a protocol for systematic review and meta-analysis. BMJ Open 2020;10:e034554. 10.1136/bmjopen-2019-034554 PMC7310998 32565455

[11] Zheng H Han Y Du Y. Regulation of hypertension for secondary prevention of stroke: the possible ‘bridging function’ of acupuncture. Complement Med Res 2018;25:45–51. 29393105 10.1159/000475930

[12] Li SK Ding DM Zhang ZL. Effects of scalp acupuncture combined with auricular point sticking on cognitive behavior ability in patients with vascular dementia. Zhongguo Zhen Jiu 2014;34:417–20. 25022106

[13] Wang LR Wang HL Lu JQ. Clinical trials of treatment of acute facial paralysis with pain by blood-letting plus acupuncture in patients. Zhen Ci Yan Jiu 2015;40:157–60. 26054203

[14] Bi JQ, Li W, Yang Q, et al. Acupuncture for the treatment of oculomotor paralysis: a pilot randomised controlled trial. Evid Based Complement Altern Med 2016, Article ID 3961450, 6 pages. 10.1155/2016/3961450 PMC4894997 27313646

[15] Zhi F, Huang Q, Zhao Y, et al. [The patterns analysis of clinical application of acupuncture for ophthalmopathy]. 10.13703/j.0255-2930.2018.08.029 30141305

[16] Rui Q. Treatment of 6 cases of oculomotor paralysis with acupuncture, 2007 Shanghai Research Institute of Acupuncture and Meridian. Zhongguo Zhen Jiu 2018 ;38:907–912.

[17] Zhou LY Zhang XM Li ZJ. Observation on therapeutic effect of eye-needling combined with medication for treatment of ophthalmoplegia. Zhongguo Zhen Jiu 2007;27:165–8. 17432638

[18] Zhang Q Ji G Cao F. Tuina for spasticity of poststroke: protocol of a systematic review and meta-analysis. BMJ Open 2020;10:e038705. 10.1136/bmjopen-2020-038705 PMC7733218 33303441

[19] Wang C Zhang X Wang D. Tuina for functional constipation: a protocol for the systematic review of randomized clinical trials. Medicine 2019;98:e14775. 30855485 10.1097/MD.0000000000014775 PMC6417547

[20] Fan Z Tian Q Guo R. Tuina for low back pain: protocol for a systematic review and meta-analysis. Medicine 2018;97:e11979. 30142831 10.1097/MD.0000000000011979 PMC6113039

[21] Shamseer L Moher D Clarke M. Preferred reporting items for systematic review and meta-analysis protocols (PRISMA-P) 2015: elaboration and explanation. BMJ 2015;350:g7647. 25555855 10.1136/bmj.g7647

[22] Guyatt GH Oxman AD Vist GE. GRADE: an emerging consensus on rating quality of evidence and strength of recommendations. BMJ 2008;336:924–6. 18436948 10.1136/bmj.39489.470347.AD PMC2335261

[23] Liu K Lin GH Zeng JC. Professor LIN Guo-hua's clinical experience of acupuncture for oculomotor nerve paralysis. Zhongguo Zhen Jiu 2020;40:1232–4. 33788494 10.13703/j.0255-2930.20191010-0001

[24] Zhao FL, Wang B, et al. “Analysis of clinical effect and prognostic factors of oculomotor nerve palsy induced by posterior communicating artery aneurysms after endovascular embolization.” Chin J Interv Radiol (Electronic Edition) 2020:151-154.

[25] Teng JY, Hu SY, et al. “Efficacy Observation on Ocular Acupuncture Combined with Mecobalamin Acupoint Injection in Traumatic Oculomotor Nerve Palsy.” World Chin Med 2019:2158-2162.

